# Candidate Sequence Variants and Fetal Hemoglobin in Children with Sickle Cell Disease Treated with Hydroxyurea

**DOI:** 10.1371/journal.pone.0055709

**Published:** 2013-02-07

**Authors:** Nancy S. Green, Katherine L. Ender, Farzana Pashankar, Catherine Driscoll, Patricia J. Giardina, Craig A. Mullen, Lorraine N. Clark, Deepa Manwani, Jennifer Crotty, Sergey Kisselev, Kathleen A. Neville, Carolyn Hoppe, Sandra Barral

**Affiliations:** 1 Department of Pediatrics, Columbia University, New York, New York, United States of America; 2 Department of Pediatrics, Yale University, New Haven, Connecticut, United States of America; 3 Department of Pediatrics, Albert Einstein College of Medicine, Bronx, New York, United States of America; 4 Department of Pediatrics, Weill Cornell University Medical School, New York, New York, United States of America; 5 Department of Pediatrics, University of Rochester, Rochester, New York, United States of America; 6 Department of Pathology and Cell Biology, Columbia University, New York, New York, United States of America; 7 Department of Pediatrics, Children’s Mercy Hospitals and Clinics, Kansas City, Missouri, United States of America; 8 Department of Hematology-Oncology, Children’s Hospital and Research Center Oakland, Oakland, California, United States of America; 9 G. H. Sergievsky Center, Columbia University, New York, New York, United States of America; University of Sao Paulo - USP, Brazil

## Abstract

**Background:**

Fetal hemoglobin level is a heritable complex trait that strongly correlates swith the clinical severity of sickle cell disease. Only few genetic loci have been identified as robustly associated with fetal hemoglobin in patients with sickle cell disease, primarily adults. The sole approved pharmacologic therapy for this disease is hydroxyurea, with effects largely attributable to induction of fetal hemoglobin.

**Methodology/Principal Findings:**

In a multi-site observational analysis of children with sickle cell disease, candidate single nucleotide polymorphisms associated with baseline fetal hemoglobin levels in adult sickle cell disease were examined in children at baseline and induced by hydroxyurea therapy. For baseline levels, single marker analysis demonstrated significant association with *BCL11A* and the beta and epsilon globin loci (*HBB* and *HBE*, respectively), with an additive attributable variance from these loci of 23%. Among a subset of children on hydroxyurea, baseline fetal hemoglobin levels explained 33% of the variance in induced levels. The variant in *HBE* accounted for an additional 13% of the variance in induced levels, while variants in the *HBB* and *BCL11A* loci did not contribute beyond baseline levels.

**Conclusions/Significance:**

These findings clarify the overlap between baseline and hydroxyurea-induced fetal hemoglobin levels in pediatric disease. Studies assessing influences of specific sequence variants in these and other genetic loci in larger populations and in unusual hydroxyurea responders are needed to further understand the maintenance and therapeutic induction of fetal hemoglobin in pediatric sickle cell disease.

## Introduction

In sickle cell disease, higher fetal hemoglobin (HbF) levels diminish de-oxygenated sickle globin polymerization in vitro [1] and reduce the incidence of disease morbidities in vivo [2], [3]. HbF is a heritable complex trait [4], [5]. Only three genetic loci have been validated as strongly associated with higher HbF in sickle cell disease: the 5′ beta globin locus (*HBB)* [5], [6], [7], [8], [9], although not the sickle mutation; the *BCL11A* repressor of HbF [5], [6], [7], [8], [10], [11]; and the *HS1L-MYB* intergenic region [5], [6], [8], [12]. Other candidate regions have not been confirmed [10], [13], [14].

Hydroxyurea is the only approved pharmacologic therapy for sickle cell disease. Its clinical and laboratory effect is understood to result largely from enhanced HbF expression [3], [15], [16], although induction occurs to a highly variable extent [3], [4], [16], [17], [18]. Children generally have higher baseline HbF levels than adults [13], [18] and a stable [15] and overall more robust HbF response to hydroxyurea [13], [18], [19]. Hydroxyurea-induced HbF is also a heritable trait [4] that generally correlates with baseline levels [18], [19], [20]. To date, only limited reports have examined relationships between hydroxyurea-induced HbF and specific genetic polymorphisms in adults with sickle cell disease [13], [21] and did not explore the recently identified major loci of interest. Genetic determinants for this clinically relevant marker of drug response have not been confirmed in children [17], [19]. Such insight would be useful for elucidating mechanisms of hydroxyurea induction and for predicting individual response.

Our multi-site observational study examined associations between baseline and hydroxyurea-induced HbF in children with sickle cell disease and candidate single nucleotide polymorphisms (SNP) in several genes associated with adult sickle cell HbF levels. Our results indicate: 1) A 33% contribution of baseline to induced levels; 2) Confirmation of single marker associations between the *HBB* and epsilon globin (*HBE)* and *BCL11A* loci and baseline HbF in children; 2) Association between *HBE* and hydroxyurea-induced HbF; 3) Additive effects of these SNPs on baseline and induced HbF in children.

## Materials

### Ethics

Studies were performed under the policies of and with specific approval from the Institutional Review Board (IRB) at Columbia University and the corresponding body at each of the other participating institutions: Yale University IRB, Albert Einstein College of Medicine IRB, Weill Cornell University Medical School IRB, University of Rochester Research Subjects Review Board, Children's Mercy Hospitals and Clinics IRB, Children’s Hospital & Research Center Oakland IRB. Written informed consent from parents of participating children and informed assent from children were obtained according to each institution’s IRB policies.

### Participants

Observational analysis of children attending sickle cell clinic was performed at five sites (see author affiliations), including prospective observation during 2010. The Oakland site provided archived data and samples. Clinical inclusion and exclusion criteria conformed to those previously described [18], [22]. Inclusion criteria are: HbSS or HbS-B^0^ thalassemia, ages 5–21 years. Exclusions criteria are: pregnancy, current or recent painful crisis, fever or other acute illness within three weeks prior to evaluation, transfusion within the prior 100 days or active transfusion therapy, abnormal elevated serum creatinine or liver transaminases. We excluded siblings to ensure genetic independence. Laboratory data at steady state represented the most recent values obtained during routine care or an average of three values from well visits over the preceding 6–12 months, if available. Percent HbF (%HbF) was determined by routine HPLC and was used as a quantitative trait at steady state for baseline and for drug-induced levels.

Duration of hydroxyurea treatment was for at least six months and was initiated for comparable clinical indications across sites (nearly all for repetitive painful crises and/or acute chest episodes). Stable hydroxyurea dosing was three months at or near maximal dose by ANC criteria, excluding data from subjects on less than 20 mg/kg/day, even if for dose-limiting toxicity. Across the six sites, drug dose averages ranged from 23.8–29 mg/kg/day and did not statistically differ between sites (F = 1.554, p = 0.210). Adherence to hydroxyurea was defined as parent report of at least 80% of prescribed doses. Laboratory data at steady state represented recent values during routine care or averaged three values from well visits over the preceding 6–12 months, if available. Percentage of HbF (HbF%) was used as a quantitative trait. Baseline HbF had not been recorded prior to treatment for nine of the 47 subjects on hydroxyurea, thus is not available. Hydroxyurea-associated increased mean red cell volume and decreased white blood cell count were used to confirm data quality. For children on hydroxyurea therapy, where available, clinical response to hydroxyurea was assessed by retrospective chart review of the number of sickle-related hospitalizations over the two-year periods preceding and while on hydroxyurea treatment.

### Description of Procedures

Twenty SNPs previously reported as associated with HbF% in sickle cell disease [6], [7], [8], [9], [11], [14], [17], [19] were genotyped (Table 1): nine in the *BCL11A* locus; two in *HBS1L-MYB* intergenic region on chromosome 6q23; three in the globin locus on chromosome 11: two 5′ sites in *HBB* (including the previously identified XmnI site [23] and one in *HBE* [19]; one in *OR51* that is upstream of *HBB* [14]; one in the glucagon-like peptide-2 receptor, *GLP2R,* found by genome-wide analysis [10]; two SNPs from the hydroxyurea-induced *SAR1A* locus [21]; one SNP each in *ARG1* and *ARG2* [19] (SNP sequences available by request).

**Table 1:** 19 SNPs associations with baseline, maximum and delta HbF%.

				Baseline HbF(N = 108)			Maximum HbF(N = 47)			Delta HbF(N = 38)			
Chr	Gene	SNP	A1	β	SE	p	β	SE	p	β	SE	p	Reference
2	*BCL11A*	rs7581162	T	0.08	0.70	0.908	0.40	1.55	0.796	−1.43	1.72	0.412	HapMap
2	*BCL11A*	rs10189857	G	0.10	0.77	0.898	0.27	1.96	0.891	3.57	2.12	0.101	6
2	*BCL11A*	rs1427407	T	2.55	0.69	* **4×10^−4^**	2.14	1.64	0.200	0.19	1.64	0.909	19
2	*BCL11A*	rs7599488	T	−0.20	0.78	0.803	0.87	1.96	0.659	4.67	2.13	0.035	6
2	*BCL11A*	rs766432	C	2.88	0.68	* **5×10^−5^**	3.38	1.59	**0.039**	1.20	1.60	0.459	10,11,19
2	*BCL11A*	rs11886868	C	2.58	0.64	* **1×10^−4^**	3.37	1.38	**0.019**	1.25	1.42	0.386	7,19
2	*BCL11A*	rs4671393	A	2.88	0.68	* **5×10^−5^**	3.38	1.59	**0.039**	1.20	1.60	0.459	7,19
2	*BCL11A*	rs7557939	G	2.51	0.64	* **2×10^−4^**	3.09	1.39	**0.031**	1.10	1.42	0.446	7,19
2	*BCL11A*	rs10184550	G	2.24	0.66	**0.001**	2.44	1.36	0.080	−0.56	1.45	0.701	11
6	*ARG1*	rs17599586	T	−0.64	1.07	0.549	−4.71	2.56	0.073	−4.43	2.42	0.076	19
6	*HBS1L-MYB*	rs28384513	C	0.24	0.90	0.792	−2.60	1.86	0.170	−1.65	1.83	0.373	7
6	*HBS1L-MYB*	rs4895441	G	−0.24	1.23	0.846	−4.91	3.06	0.116	−1.71	3.20	0.595	7
10	*SAR1A*	rs2310991	A	0.05	0.62	0.942	−0.60	1.61	0.711	0.03	1.62	0.984	21
11	*HBB*	rs10128556	T	2.63	1.34	0.057	5.25	2.86	0.074	2.53	2.62	0.342	6
11	*HBB*	rs7482144	A	3.88	0.99	* **2×10^−4^**	7.61	3.97	0.062	2.69	3.67	0.468	7
11	*HBE*	rs7130110	C	2.86	0.68	* **6×10^−5^**	7.82	2.07	* **5×10^−4^**	6.04	1.97	**0.004**	19
11	*OR51B6*	rs5024042	A	1.70	0.78	**0.031**	3.04	1.93	0.122	1.68	1.85	0.371	13
14	*ARG2*	rs2295644	A	1.13	0.76	0.142	−0.04	1.79	0.983	−0.01	1.91	0.995	19
17	*GLP2R*	rs12103880	A	0.68	0.69	0.324	−3.66	1.30	**0.008**	−2.74	1.46	0.068	10

Bold indicates SNPs reaching nominally significance (p≤0.05).

* Indicates significant SNP after Bonferroni correction for multiple testing.

SNP marker genotyping of minor allele frequencies ranging from 0.07 to 0.45 was performed using Sequenom MassArray iPLEX platform with matrix-assisted laser desorption/ionization time of flight mass spectrometry (Sequenom, San Diego, CA). PCR assays and mass extension reactions were designed using mass array assay design software (Sequenom). PCR assays used Applied Biosystems Geneamp PCR thermocyclers (Foster City, CA) and analyzed by mass array compact mass spectrometer (Bruker Daltonik, Billerica, MA) and Spectro TYPER software (Sequenom). (SNP sequences, PCR and analytic conditions are available upon request.) Genotyping was performed in duplicate with separate assays, with genotype frequency distribution at each SNP tested for deviations from Hardy-Weinberg equilibrium. Accurate genotyping of *SAR1A* SNP rs4282891 required sequencing. The sickle genotype, rs334, was assayed to confirm the diagnosis for each sample.

### Statistical Methods

Relationships between each of the three quantitative HbF values were assessed by Pearson correlation analysis. Genetic associations were assessed for candidate SNPs with %HbF at baseline, on hydroxyurea treatment (“maximum HbF”), and the hydroxyurea-induced increment over baseline (“delta HbF”) (Table 1). For each HbF value, quantitative associations with a dose effect model of the minor allele were tested using a linear regression analysis, adjusted for sex and age. Baseline HbF levels were log10 transformed to fit a normal distribution; maximum and delta HbF were normally distributed. Significance is reported for nominal p-values of 0.05 or less, and with Bonferroni adjustment of p-values for multiple testing (adjusted p-value of 3×10^−3^ for the 19 SNPs tested – see below). Given the fixed sample size, power was estimated based on the smallest detectable differences in the average levels of baseline and hydroxyurea-induced fetal hemoglobin HbF. Power was computed using QUANTO software (http://hydra.usc.edu/gxe/) assuming an additive allele effects model, an alpha threshold of 0.05, a range of different values for effect sizes (β) and SNP allele frequency (Figure S2) [24].

Limited number of subjects of each genotype precluded analysis of potential dose effect of homozygotes. Trend analysis was performed by analysis of variance tested for individual and additive effects for each allele. Percentage of variance attributable to SNPs in *BCL11A*, *HBB* and *HBE* was estimated using linear regression analysis comparing models consisting of age, sex, individual SNPs and multiple SNPs with a reduced model consisting of sex and age (SPSS Inc., Chicago, IL). To evaluate whether *BCL11A* haplotypes was in closer linkage disequilibrium with causal variants than single SNPs, haplotype association analysis was performed with PLINK software [25] using a haplotype block of four *BCL11A* SNPs in strong linkage disequilibrium (r^2^>0.70) [6].

## Results

### Clinical

117 children from the six sites met study criteria (Data from each site are shown in Table S1). The mean age and standard deviation was 12.5 (SD = 4.9), with 52% male. Six subjects (5.1%) had HbS-B^0^ thalassemia (Table S1A). A subset of children (N = 47) was on hydroxyurea, 38 of whom had recorded baseline and maximum HbF values. Hydroxyurea dosing ranged from 20–30.7 mg/kg/day, averaging 25.3 (SD = 3.0) mg/kg/day. During the two years prior to hydroxyurea therapy, children (N = 22 with available data) had 0–13 hospital admissions (mean = 3.7, SD = 2.8); only one child had no hospitalizations. During a two year period while on hydroxyurea therapy, these same children had 0–6 hospitalizations (mean = 1.4,SD = 1.5). While on hydroxyurea, nearly all had fewer hospitalizations over a two year period (p = 0.001), and 32% had none.

**Figure 1:**
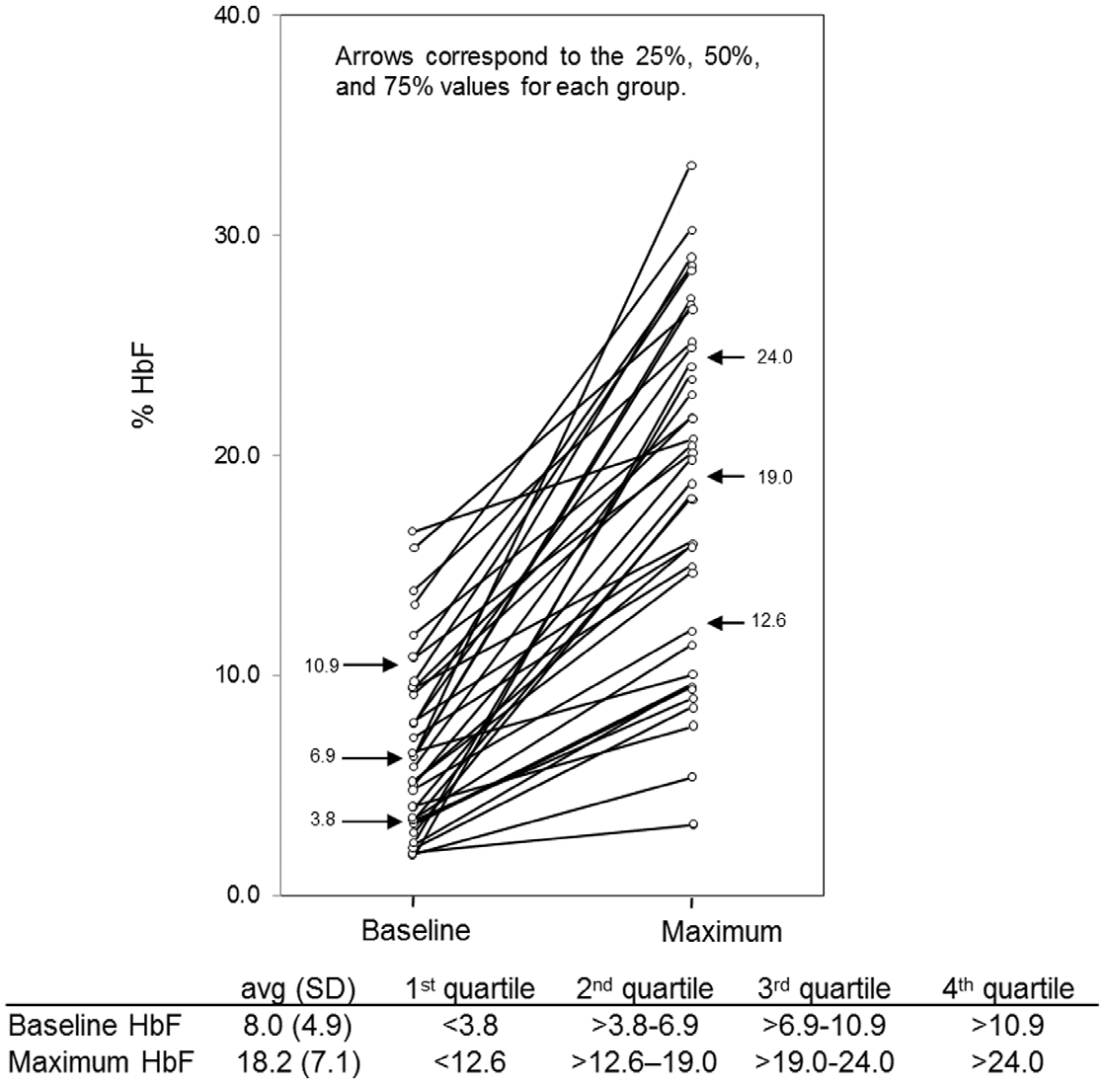
Baseline and hydroxyurea-induced maximum HbF for each subject (comparable to References 18, 19, 26).

Mean HbF% values were: baseline HbF 8.0 (SD = 4.9, N = 108), maximum HbF 18.2 (SD = 7.1, N = 47), and delta HbF 11.6 (SD = 6.3, N = 38). Among those subjects with complete data for baseline and hydroxyurea-induced HbF (N = 38), distributions of baseline and induced HbF were comparable to those previously reported for children on hydroxyurea (Figure 1, Figure S1) [18] thereby validating our observational data. No significant effect of gender was detected for any of the three quantitative traits; age appeared to have some influence on baseline HbF. Mean baseline HbF at each site ranged from 6.8 (SD = 5.0) to 9.8 (SD = 5.6), with no significant difference in mean values (F = 0.808, p = 0.523), excluding the average value of 12.9% from Rochester (N = 8). At the three sites with at least four subjects on hydroxyurea (Columbia, Yale and Oakland), no differences were found in induced maximum or delta HbF (F = 0.668, p = 0.418 and F = 0.404, p = 0.530, respectively).

Pearson correlation for the 38 children with complete baseline and hydroxyurea-induced HbF demonstrated high levels of relatedness for: baseline and induced maximum HbF levels (r^2^ = 0.59, p<0.001), similar to previously reported [18]; maximum and delta HbF (r^2^ = 0.86, p<0.001); but not between baseline and delta HbF (r^2^ = 0.08, p = 0.632). Those in the lowest quartile for baseline levels were highly likely to remain in the lowest quartile (Q1) for induced HbF (r2 = 0.565, p<0.001) (Figure 1). Only two children (5.3%) were induced from the lowest to highest quartiles (Q3+Q4), and did not appear to differ by age, sex, sickle type, hydroxyurea dose, genotype, or other discernible variable, although the small number precluded statistical analysis. By linear regression analysis adjusted for age and sex, overall percentage of variance in hydroxyurea-induced HbF attributable to baseline levels was 33%.

### Genetic

Call rates uniformly exceeded 97% for each SNP. One *SAR1A* gene SNP, rs428289, [21] was monomorphic in subjects treated with hydroxyurea, precluding analysis of variants. Genotyping of the other 19 SNPs revealed minor allele frequencies that were comparable across the six sites (Table S1B) and to allele frequencies among sickle cell populations in the U.S and elsewhere [7], confirming validity of our pooling strategy. Log_10_ transformation of baseline HbF had no effect on SNP associations; non-transformed results are shown for comparison with induced values (Table 1).

#### Baseline HbF

Given the fixed sample size and SNP allele frequencies, the study had 90% power to detect differences in average levels of baseline HbF% between 2.0 and 2.5 (Figure S2A). Single marker SNP analysis revealed several significant associations to baseline HbF (Table 1). One of two SNPs tested within the *HBB* locus, rs7482144 [7], and the *HBE* SNP [19] were significantly associated with baseline HbF after Bonferroni correction. Six *BCL11A* SNPs were significantly associated with baseline HbF, of which five withstood correction for multiple comparisons, including the intronic variant, rs4671393 [6], [7]. To test for closer linkage disequilibrium with causal variants within the *BCL11A* haplotype block than single SNPs [6], analysis revealed several common haplotypes (frequency = 0.05), with statistical association and effect size comparable to those seen with single marker analysis (data not shown). Power was insufficient to detect effects sizes of <3, such as for markers in the HBS1L-MYB intergenic region (Table 1).

Trend analysis using the SNP allele associated with higher baseline HbF values (“favorable allele”) in one or both of the *BCL11A* (rs4671393) and *HBE* (rs7130110) loci demonstrated a statistically significant additive effect of each SNP and a two-fold difference for both (p_trend_<0.004) (Table 2). The *HBB* marker (rs7482144) had effects identical to those of *HBE* (not shown). By linear regression analysis of individual markers accounting for age and sex, the percentage of baseline HbF variance attributable each to the *BCL11A* or *HBE* SNP was 13%, and 10% for *HBB* (Table S2A). In the multiple marker model, the *BCL11A* SNP combined with either globin marker had an additive effect to 21–23%. Contributions from *HBE* and *HBB* appeared to be redundant. These two markers are in strong linkage disequilibrium within the globin locus (r2 = 0.86, p<0.001), with different prevalence of the minor allele: 18% for *HBE* and 12% for *HBB.*

**Table 2:** Incremental effect of allelotypes in *BCL11A* and *HBE* on Baseline and Maximum HbF%.

Allelotype					
*BCL11A*	*HBE*	N (108)	Avg Baseline	N (47)	Avg Maximum
rs4671393	rs7130110		HbF% (SD)		HbF% (SD)
+	+	16	11.8 (5.2)		
−	+	29	7.9 (5.3)	23#	20.8 (7.5)**
+	−	18	8.8 (4.7)		
−	−	45	6.4 (3.7)*	24	15.5 (5.6)

Baseline HbF%: Trend analysis across allelotypes: F(df,3) = 8.48, p_trend_ = 0.004.

Maximum HbF%: Trend analysis across 0 or ≥1 A alleles: F(df,2) = 7.43, p_trend_ = 0.009.

The + denotes minor allele (A) associated with higher HbF%; the - denotes the other allele (G),

with the *BCL11A* A or *HBE* allele in 1 or 2 copies.

* Reference allelotype.

** Not significant when adjusted for baseline HbF.

# Includes subjects with either or both minor A alleles.

#### Hydroxyurea-induced HbF

The study had ≥80% power to detect SNPs with effect sizes larger than 2.5 with allele frequencies of 0.25 or higher; estimated power decreased with lower SNP allele frequencies (Figure S2B). Among all single markers tested, only the *HBE* SNP [19] was significantly associated with maximum HbF after correction for multiple testing, and remained nominally associated even after adjusting for baseline HbF (p = 0.001) (Table 1). This same SNP was also unique in being associated with the hydroxyurea-induced increment, delta HbF (p = 0.004). Nominal associations were also found between maximum HbF and four of the same six *BCL11A* SNPs associated with baseline HbF (Table 1). However, no association of *BCL11A* single marker with maximum HbF withstood adjustment for baseline HbF (Table 1). Haplotype analysis of *BCL11A* indicated nominal association with hydroxyurea-induced HbF, remaining at borderline significant even after adjustment for baseline HbF (data not shown).

By trend analysis, subjects with a favorable allele in at least one of the *BCL11A* or *HBE* loci had higher average values, 20.8 (SD = 7.5, N = 23), compared to 15.5 (SD = 5.6, N = 24) for those without either allele (p_trend_ = 0.009) (Table 2). As with single SNP analysis, statistical significance of this trend analysis did not withstand adjustment for baseline fetal hemoglobin (p = 0.0.086). As seen for baseline HbF, the *HBB* marker (rs7482144) had effects identical to those of *HBE* (p_trend_ = 0.018, not shown). By linear regression analysis of the three markers, alone or in combination, only the *HBE* SNP independently and significantly added to the phenotypic variance contributed by the baseline levels. In combination, baseline level (33%) and the *HBE* (13%, p<0.001) contributed an estimated 46% of the variance (Table S2B).

The *GLP2R* allele, not associated with baseline HbF, was nominally associated with maximum HbF; the *SAR1A* SNP, rs2310991, [21] was not.

## Discussion

Children with sickle cell disease generally have higher baseline HbF levels than adults and more pronounced HbF response to hydroxyurea [5], [6], [7], [8], [9], [11], [13], [16], [18], [19], [20], [26]. Findings from this multi-site, observational analysis corroborate in children the correlation between baseline and hydroxyurea-induced HbF levels [18], [19], and demonstrate that one third of the variance in induced HbF is attributable to baseline levels. In contrast to the induced HbF, the treatment-associated increment appears to be a less relevant marker, as baseline and delta levels did not correlate and those with high baseline more likely have highly inducible HbF%.

For baseline HbF, we confirm in children the associations between single candidate markers in the *BCL11A* and *HBB* or *HBE* loci [19]. Comparable marker contributions to baseline HbF were reported for *BCL11A* and *HBB* in adult sickle cell disease [6], [7]. In our analysis, statistical effects on HbF from *BCL11A* and either globin marker were additive, and were associated with two-fold higher levels for patients with favorable alleles in both loci. An apparent redundant influence exists between *HBB* and *HBE* markers, suggesting a statistical effect stemming from tight linkage and/or overlapping biologic effects.

For hydroxyurea-induced HbF, the *HBE* marker was the most robustly associated with drug-induced HbF levels, an effect that was independent of and additive to baseline levels. Despite a sample size limiting statistical power for individual SNPs, trend analysis with *BCL11A* and *HBE* or *HBB* markers suggests substantially higher induced levels associated with one or both favorable alleles. In combination with baseline levels, the *HBE* accounted for almost half of the variance of induced levels. Taken together, these data support a model of overlapping genetic regulation of HbF in pediatric SCD for both steady states [17].

Roles of other SNP markers are less clear. Nominal association with *GLP2R* for hydroxyurea-induced HbF, but not baseline, will need confirmation and biologic rationale. No associations were detected with SNPs in other candidate loci such as *HBS1L-MYB*, presumably from insufficient power and its lesser impact on baseline HbF in sickle cell disease [6]. Another hematologic parameter, alpha-thalassemia status, is not independently associated with hydroxyurea-induced HbF in children [19]. Effects of the drug’s pharmaco-kinetics on HbF response are unclear [19].

Limitations: Study limitations include sample size and observational assessment, where therapeutic approaches to hydroxyurea and drug adherence may vary among sites despite inclusion criteria. Nonetheless, hydroxyurea dose and distribution of drug-induced HbF were comparable to those from prospective pediatric hydroxyurea studies [18]. Moreover, a threshold hydroxyurea dose of 20 mg/kg/day was employed to reduce HbF variability from low drug dosing.

Clinically, HbF level is arguably the strongest predictor of disease severity [2], [3]. Baseline HbF in children remains the best forecaster of hydroxyurea-induced HbF but accounts for only a portion of drug response [19]. Despite a modest sample size, clinically relevant differences in HbF [2], [3] in our study population appeared to be associated with specific alleles in three genetic loci. These findings suggest that induced levels in pediatric disease share some of the major regulatory loci associated with baseline levels. Our *HBE* marker data also suggest that induced levels may reflect effects that are additive to those of baseline. In summary, our findings suggest that baseline and hydroxyurea-induced HbF likely are influenced by these genetic loci, while distinct genetic, pharmacologic, and clinical determinants also exist [18], [19]. Genetic studies examining larger pediatric populations on hydroxyurea and unusual responders are needed to assess the specific sequence variants in these and other genetic loci responsible for HbF response.

## Supporting Information

Figure S1 **Histograms for Baseline, Maximum and Delta HbF%.**
(JPG)

Figure S2 **Power analyses based on minor allele frequencies.** S2A. Baseline HbF. S2B. Maximum HbF
(DOC)

Table S1 **Clinical and Genetic Data by Study Site.** S1A. Clinical data by study site. S1B. Minor allele frequencies by study site.
(DOC)

Table S2 **HbF linear regression analysis to compare full model consisting of age, sex, individual and multiple SNPs.** S2A. Baseline HbF%. S2B. Maximum HbF%
(DOC)
