# Supplementary figures and images for: Candidate Sequence Variants and Fetal Hemoglobin in Children with Sickle Cell Disease Treated with Hydroxyurea
Source: PLoS One. 2013 Feb 7;8(2):e55709. doi: 10.1371/journal.pone.0055709 (PMC3567082; doi:10.1371/journal.pone.0055709)

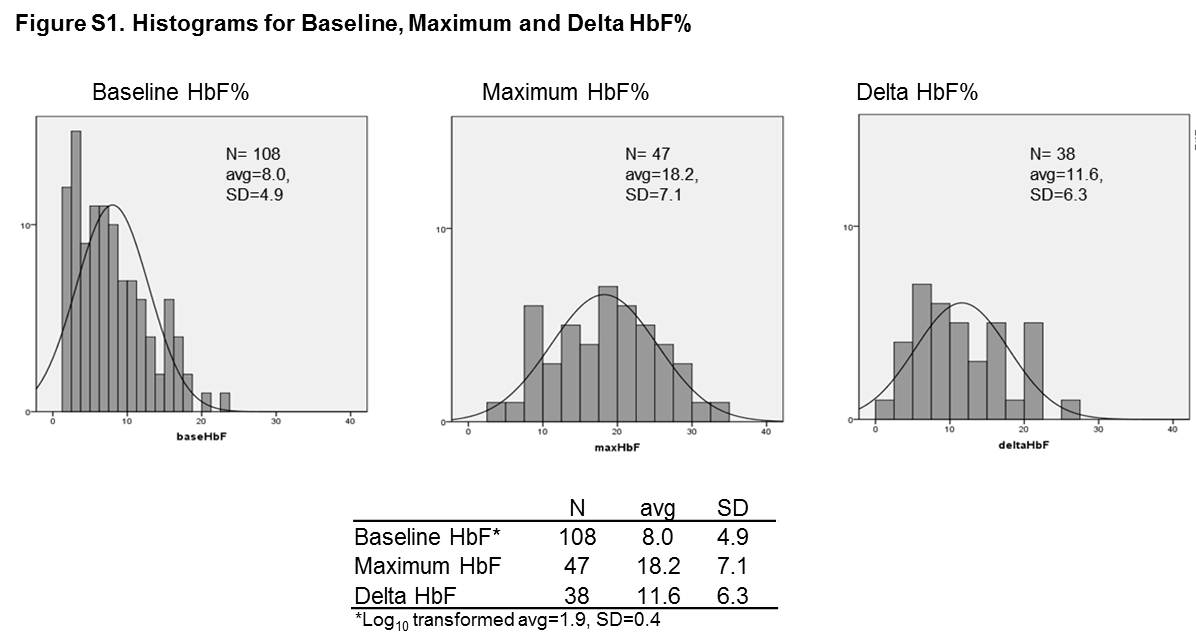

Supplement: Figure S1 — Histograms for Baseline, Maximum and Delta HbF%. (JPG) [file pone.0055709.s001.jpg]
