# Supplementary material for: Candidate Sequence Variants and Fetal Hemoglobin in Children with Sickle Cell Disease Treated with Hydroxyurea
Source: PLoS One. 2013 Feb 7;8(2):e55709. doi: 10.1371/journal.pone.0055709 (PMC3567082; doi:10.1371/journal.pone.0055709)
Supplement: Figure S2 — Power analyses based on minor allele frequencies. S2A. Baseline HbF. S2B. Maximum HbF (DOC) [file pone.0055709.s002.doc]

| **Figure S2. Power analyses based on minor allele frequencies**.  Figure S2A. Power analysis for Baseline HbF%. | | | | | |  |  |  |  |  |
| --- | --- | --- | --- | --- | --- | --- | --- | --- | --- | --- |
|  |  |  |  |  |  |  |  |  |  |  |
|  |  |  |  |  |  | Levels of Baseline HbF for risk | | | |  |
|  |  |  |  |  |  | allele dosage | |  |  |  |
|  |  |  |  | avg allele freq | | 1 or 2 copies | | none |  |  |
| SNP | Gene | allele | max β | min | max | avg | SD | avg | SD |  |
| rs7482144 | *HBB* | A | 3.88 | 0.06 | 0.22 | 10.9 | 5.3 | 7.1 | 4.4 |  |
| rs4671393 | *BCL11A* | A | 2.88 | 0.21 | 0.27 | 11.1 | 5.2 | 7.0 | 4.4 |  |
| rs7130110 | *HBE* | C | 2.86 | 0.11 | 0.28 | 11.7 | 4.0 | 7.0 | 4.4 |  |
|  |  |  |  |  |  |  |  |  |  |  |
| 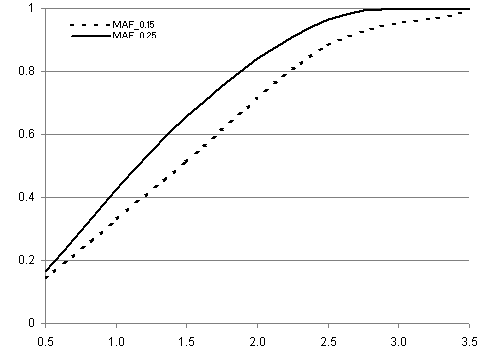 Power  βg  βg corresponds to the main genetic effect, the change in the average of Baseline HbF per increase in the risk allele dose   |  | | --- | |  |  |  |  |  |  |  |  |  |  |
|  |  |  |  |  |  |  |  |  |  |  |
|  |  |  |  |  |  |  |  |  |  |  |
|  |  |  |  |  |  |  |  |  |  |  |
|  |  |  |  |  |  |  |  |  |  |  |
|  |  |  |  |  |  |  |  |  |  |  |
|  |  |  |  |  |  |  |  |  |  |  |
|  |  |  |  |  |  |  |  |  |  |  |
|  |  |  |  |  |  |  |  |  |  |  |
|  |  |  |  |  |  |  |  |  |  |  |
|  |  |  |  |  |  |  |  |  |  |  |
|  |  |  |  |  |  |  |  |  |  |  |
|  |  |  |  |  |  |  |  |  |  |  |
|  |  |  |  |  |  |  |  |  |  |  |
|  |  |  |  |  |  |  |  |  |  |  |
|  |  |  |  |  |  |  |  |  |  |  |
|  |  |  |  |  |  |  |  |  |  |  |
|  |  |  |  |  |  |  |  |  |  |  |
|  |  |  |  |  |  |  |  |  |  |  |
|  |  |  |  |  |  |  |  |  |  |  |
|  |  |  |  |  |  |  |  |  |  |  |
|  |  |  |  |  |  |  |  |  |  |  |
|  |  |  |  |  |  |  |  |  |  |  |
|  |  |  |  |  |  |  |  |  |  |  |

| Figure S2B. Power analysis for Maximum HbF%. | | | | | |  |  |  |  |  |
| --- | --- | --- | --- | --- | --- | --- | --- | --- | --- | --- |
|  |  |  |  |  |  |  |  |  |  |  |
|  |  |  |  |  |  | Levels of Maximum HbF for risk | | | |  |
|  |  |  |  |  |  | allele dosage | |  |  |  |
|  |  |  |  | avg allele freq | | 1 or 2 copies | | none |  |  |
| SNP | Gene | allele | max β | min | max | avg | SD | avg | SD |  |
| rs7482144 | *HBB* | A | 7.61 | 0.06 | 0.22 | 27.0 | 5.7 | 17.1 | 6.7 |  |
| rs4671393 | *BCL11A* | A | 3.38 | 0.21 | 0.27 | 25.4 | 3.4 | 17.4 | 6.7 |  |
| rs7130110 | *HBE* | C | 7.82 | 0.11 | 0.28 | 27.4 | 4.0 | 16.7 | 6.5 |  |
|  |  |  |  |  |  |  |  |  |  |  |
| βg corresponds to the main genetic effect, the change in the average of Maximum HbF per increase in the risk allele dose  βg  Power 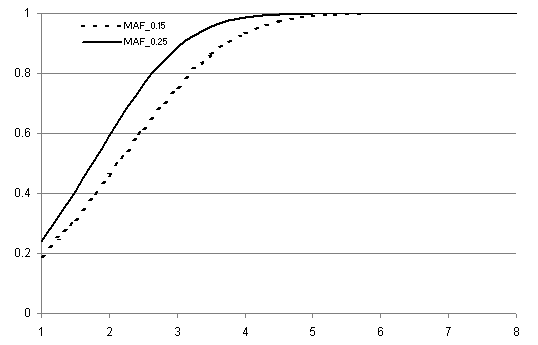  |  | | --- | |  |  |  |  |  |  |  |  |  |  |
|  |  |  |  |  |  |  |  |  |  |  |
|  |  |  |  |  |  |  |  |  |  |  |
|  |  |  |  |  |  |  |  |  |  |  |
|  |  |  |  |  |  |  |  |  |  |  |
|  |  |  |  |  |  |  |  |  |  |  |
|  |  |  |  |  |  |  |  |  |  |  |
|  |  |  |  |  |  |  |  |  |  |  |
|  |  |  |  |  |  |  |  |  |  |  |
|  |  |  |  |  |  |  |  |  |  |  |
|  |  |  |  |  |  |  |  |  |  |  |
|  |  |  |  |  |  |  |  |  |  |  |
|  |  |  |  |  |  |  |  |  |  |  |
|  |  |  |  |  |  |  |  |  |  |  |
|  |  |  |  |  |  |  |  |  |  |  |
|  |  |  |  |  |  |  |  |  |  |  |
|  |  |  |  |  |  |  |  |  |  |  |
|  |  |  |  |  |  |  |  |  |  |  |
|  |  |  |  |  |  |  |  |  |  |  |
|  |  |  |  |  |  |  |  |  |  |  |
|  |  |  |  |  |  |  |  |  |  |  |
|  |  |  |  |  |  |  |  |  |  |  |
|  |  |  |  |  |  |  |  |  |  |  |
