# Supplementary material for: Candidate Sequence Variants and Fetal Hemoglobin in Children with Sickle Cell Disease Treated with Hydroxyurea
Source: PLoS One. 2013 Feb 7;8(2):e55709. doi: 10.1371/journal.pone.0055709 (PMC3567082; doi:10.1371/journal.pone.0055709)
Supplement: Table S1 — Clinical and Genetic Data by Study Site. S1A. Clinical data by study site. S1B. Minor allele frequencies by study site. (DOC) [file pone.0055709.s003.doc]

Table S1. Clinical and Genetic Data by Study Site.

Table S1A. Clinical data by study site.

Table S1B. Minor allele frequencies by study site.

|  |  |  | Cornell |  | Columbia | | Einstein | | Oakland | | Rochester | | Yale |  |
| --- | --- | --- | --- | --- | --- | --- | --- | --- | --- | --- | --- | --- | --- | --- |
|  |  |  | Nchr=18 | | Nchr=48 | | Nchr=38 | | Nchr=54 | | Nchr=16 | | Nchr=60 | |
| Chr | Gene | SNP | A1 | MAF | A1 | MAF | A1 | MAF | A1 | MAF | A1 | MAF | A1 | MAF |
| 2 | *BCL11A* | rs7581162 | T | 0.22 | A | 0.40 | A | 0.47 | T | 0.40 | T | 0.25 | T | 0.45 |
| 2 | *BCL11A* | rs10189857 | G | 0.50 | G | 0.29 | G | 0.29 | G | 0.30 | G | 0.44 | G | 0.30 |
| 2 | *BCL11A* | rs1427407 | T | 0.22 | T | 0.23 | T | 0.18 | T | 0.22 | T | 0.13 | T | 0.22 |
| 2 | *BCL11A* | rs7599488 | T | 0.50 | T | 0.27 | T | 0.29 | T | 0.30 | T | 0.31 | T | 0.30 |
| 2 | *BCL11A* | rs766432 | C | 0.22 | C | 0.27 | C | 0.21 | C | 0.25 | C | 0.25 | C | 0.22 |
| 2 | *BCL11A* | rs11886868 | C | 0.22 | C | 0.29 | C | 0.21 | C | 0.33 | C | 0.38 | C | 0.28 |
| 2 | *BCL11A* | rs4671393 | A | 0.22 | A | 0.27 | A | 0.21 | A | 0.25 | A | 0.25 | A | 0.22 |
| 2 | *BCL11A* | rs7557939 | G | 0.22 | G | 0.29 | G | 0.21 | G | 0.33 | G | 0.38 | G | 0.27 |
| 2 | *BCL11A* | rs10184550 | G | 0.11 | G | 0.40 | G | 0.45 | G | 0.27 | G | 0.44 | G | 0.30 |
| 6 | *ARG1* | rs17599586 | T | 0.11 | T | 0.08 | T | 0.11 | T | 0.09 | T | 0.19 | T | 0.13 |
| 6 | *HBS1L-MYB* | rs28384513 | C | 0.11 | C | 0.13 | C | 0.16 | C | 0.25 | C | 0.19 | C | 0.17 |
| 6 | *HBS1L-MYB* | rs4895441 | G | 0.11 | G | 0.00 | G | 0.05 | G | 0.13 | G | 0.06 | G | 0.08 |
| 10 | *SAR1A* | rs2310991 | C | 0.33 | A | 0.48 | A | 0.44 | A | 0.44 | A | 0.50 | C | 0.48 |
| 11 | *HBB* | rs10128556 | T | 0.33 | T | 0.10 | T | 0.00 | T | 0.03 | T | 0.00 | T | 0.00 |
| 11 | *HBB* | rs7482144 | A | 0.22 | A | 0.10 | A | 0.08 | A | 0.06 | A | 0.13 | A | 0.17 |
| 11 | *HBE* | rs7130110 | C | 0.28 | C | 0.19 | C | 0.13 | C | 0.11 | C | 0.19 | C | 0.23 |
| 11 | *OR51B6* | rs5024042 | A | 0.28 | A | 0.17 | A | 0.18 | A | 0.13 | A | 0.19 | A | 0.15 |
| 14 | *ARG2* | rs2295644 | A | 0.28 | A | 0.31 | A | 0.37 | A | 0.35 | A | 0.31 | A | 0.37 |
| 17 | *GLP2R* | rs12103880 | A | 0.44 | A | 0.31 | A | 0.37 | A | 0.46 | A | 0.50 | A | 0.48 |
| Nchr corresponds to the number of chromosomes; A1 corresponds to the minor allele name based on whole sample; MAF stands fro minimum allele frequency | | | | | | | | | | | | | | |
