# Supplementary material for: Candidate Sequence Variants and Fetal Hemoglobin in Children with Sickle Cell Disease Treated with Hydroxyurea
Source: PLoS One. 2013 Feb 7;8(2):e55709. doi: 10.1371/journal.pone.0055709 (PMC3567082; doi:10.1371/journal.pone.0055709)
Supplement: Table S2 — HbF linear regression analysis to compare full model consisting of age, sex, individual and multiple SNPs. S2A. Baseline HbF%. S2B. Maximum HbF% (DOC) [file pone.0055709.s004.doc]

Table S2. HbF linear regression analysis to compare full model consisting of age, sex, individual and multiple SNPs.

Table S2A. Baseline HbF%.

|  |  |  |  |  |  | bootstrap β coefficients | |  |
| --- | --- | --- | --- | --- | --- | --- | --- | --- |
|  |  |  |  |  |  | for the SNP markers | |  |
|  |  | adjR^2^ | SE | %deltaR^2^ | sig | β | 95%CI |  |
| **Reduced model** | **age+sex** | **0.06** | 4.72 |  | 0.014 |  |  |  |
| individual SNPs | *BCL11A* | 0.19 | 4.37 | 13.0 | 0.000 | 2.89 | 1.16, 4.41 |  |
|  | *HBB* | 0.16 | 4.45 | 10.0 | 0.000 | 3.69 | 1.67, 5.81 |  |
|  | *HBE* | 0.19 | 4.39 | 13.0 | 0.000 | 2.86 | 1.54, 3.95 |  |
| multiple SNPs | *BCL11A+HBB* | 0.27 | 4.15 | 21.0 | 0.000 | 2.62 | 0.92, 4.05 |  |
|  |  |  |  |  |  | 3.24 | 1.33, 5.22 |  |
|  | *HBB+HBE* | 0.18 | 4.40 | 12.0 | 0.000 | 0.83 | -3.60, 4.79 |  |
|  |  |  |  |  |  | 2.36 | 0.05, 5.05 |  |
|  | *BCL11A+HBE* | 0.29 | 4.10 | 23.2 | 0.000 | 2.56 | 0.95, 4.19 |  |
|  |  |  |  |  |  | 2.52 | 1.13, 3.66 |  |
| all 3 SNPs | *BCL11A+HBB*+*HBE* | 0.29 | 4.11 | 23.0 | 0.000 | 2.56 | 0.75, 4.02 |  |
|  |  |  |  |  |  | 0.77 | -2.66, 3.90 |  |
|  |  |  |  |  |  | 2.06 | -0.08, 4.23 |  |
| Significance corresponds to the p-value for the ANOVA test, the significance of the models comparison; if sig < 0.05, then the model is significant at 95% signficance level | | | | | | | | |
| adjR^2^ measures the proportion of the variance in baseline HbF that was explained by variations in the independent variables | | | | | |  |  |  |
| deltaR^2^ is computed by sustracting adjR^2^ of the specific model to the adjR^2^ of the reduced model of age and sex | | | | |  |  |  |  |
| The reported SNPs β coefficients and confidence intervals correspond to bootstrap estimates (1000 samples, 95% significance level). | | | | | | |  |  |

Table S2B. Maximum HbF%

|  |  |  |  |  |  |  | bootstrap β coefficients |  |
| --- | --- | --- | --- | --- | --- | --- | --- | --- |
|  |  |  |  | %deltaR^2^ |  |  | for the SNP markers | |
|  |  | adjR^2^ | SE | model 1 | model 2 | sig | β | 95%CI |
| **Reduced model 1** | **age+sex** | **0.06** | 4.72 |  |  | 0.014 |  |  |
|  | **baseline HbF** | 0.32 | 6.34 |  |  | 0.000 |  |  |
| **Reduced model 2** | **age+sex+baseline** | **0.33** | 6.33 |  |  | 0.001 |  |  |
| individual SNPs | age+sex+baseline+*BCL11A* | 0.31 | 6.42 | 25.0 | -2.0 | 0.003 | 0.40 | -3.37, 3.67 |
|  | age+sex+baseline+*HBB* | 0.36 | 6.18 | 30.0 | 3.0 | 0.001 | 5.87 | -1.38, 13.47 |
|  | age+sex+baseline+*HBE* | 0.46 | 5.65 | 40.0 | 13.0 | 0.000 | 6.34 | 2.32, 11.69 |
| multiple SNPs | age+sex+baseline+*BCL11A* | 0.34 | 6.27 | 28.0 | 1.0 | 0.002 | 0.17 | -3.36, 3.56 |
|  | +*HBB* | |  |  |  |  | 5.85 | -2.79, 15.46 |
|  | age+sex+baseline+*HBB*+*HBE* | 0.46 | 5.68 | 40.0 | 13.0 | 0.000 | -3.74 | -15.79, 8.08 |
|  |  | |  |  |  |  | 8.09 | 1.99, 13.16 |
|  | age+sex+baseline+*BCL11A*+*HBE* | 0.45 | 5.74 | 38.8 | 11.8 | 0.000 | -0.08 | -3.92, 3.05 |
|  |  | |  |  |  |  | 6.36 | 2.20, 12.05 |
| all 3 SNPs | age+sex+baseline+*BCL11A* | 0.44 | 5.78 | 38.0 | 11.0 | 0.000 | -0.07 | -3.79, 3.05 |
|  | +*HBB*+*HBE* | |  |  |  |  | -3.74 | -16.08, 6.95 |
|  |  |  |  |  |  |  | 8.10 | 2.23, 13.37 |
